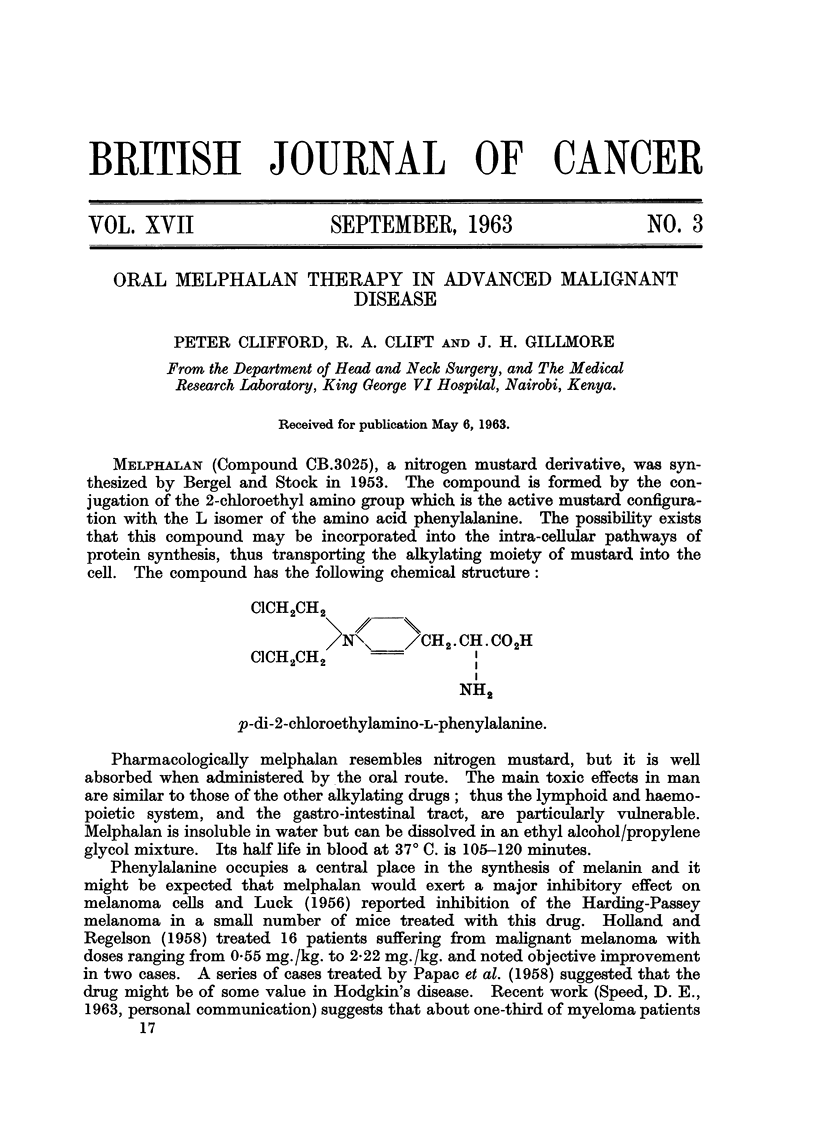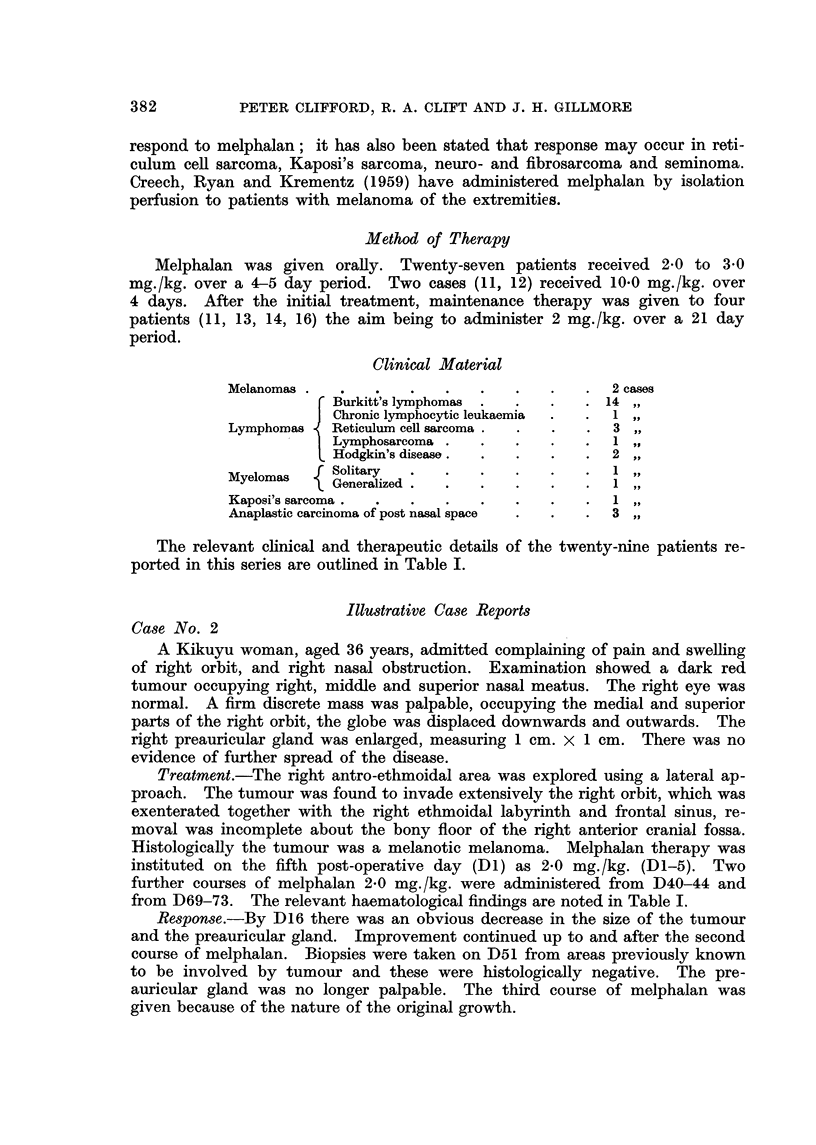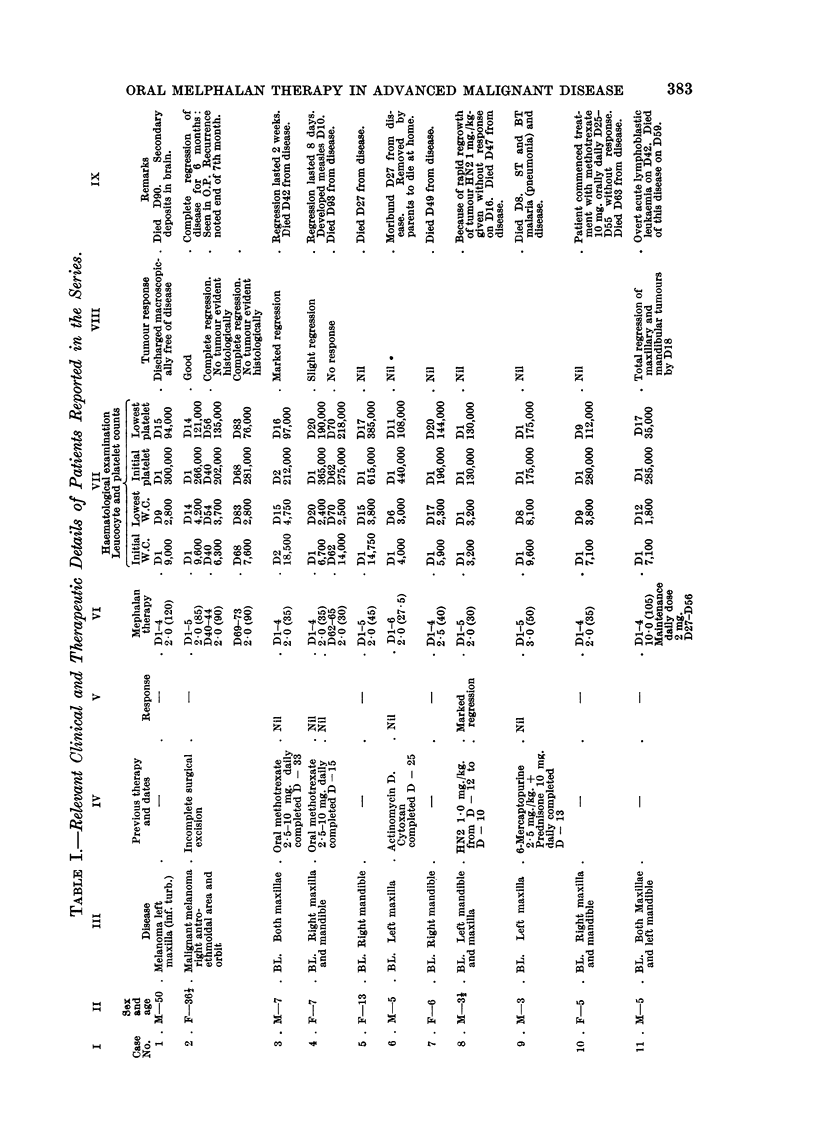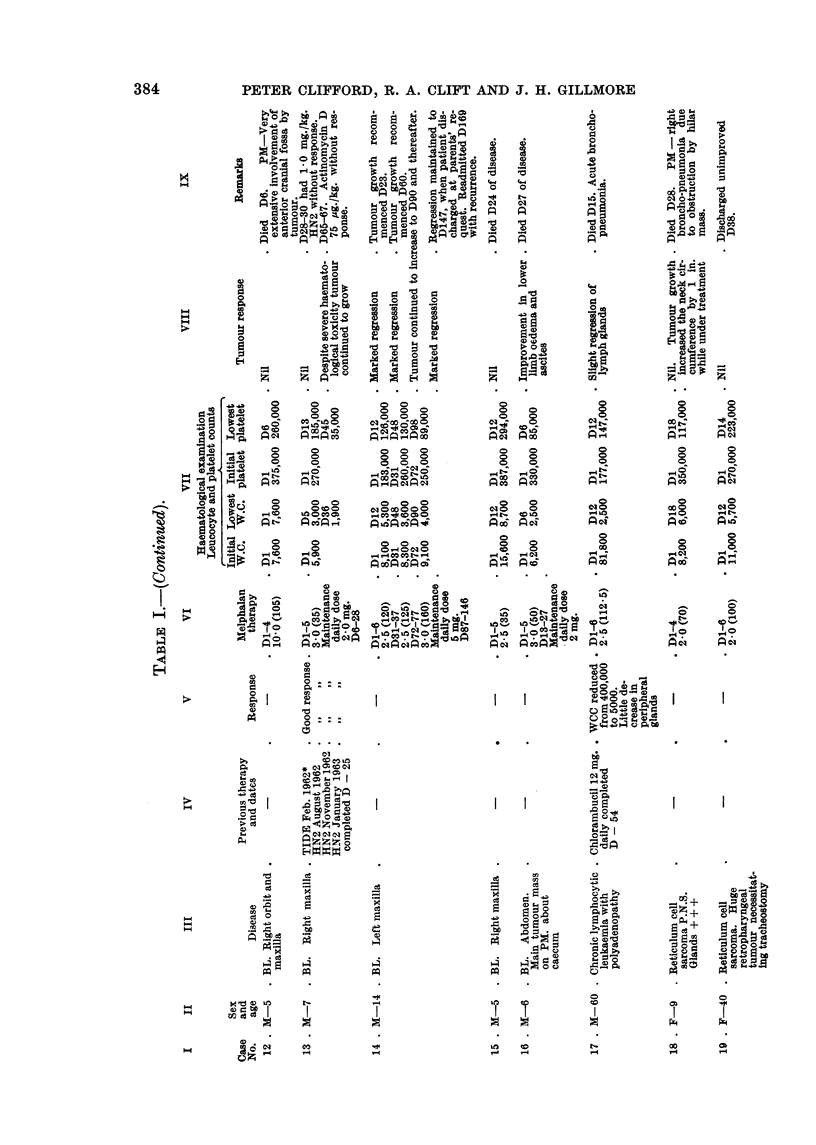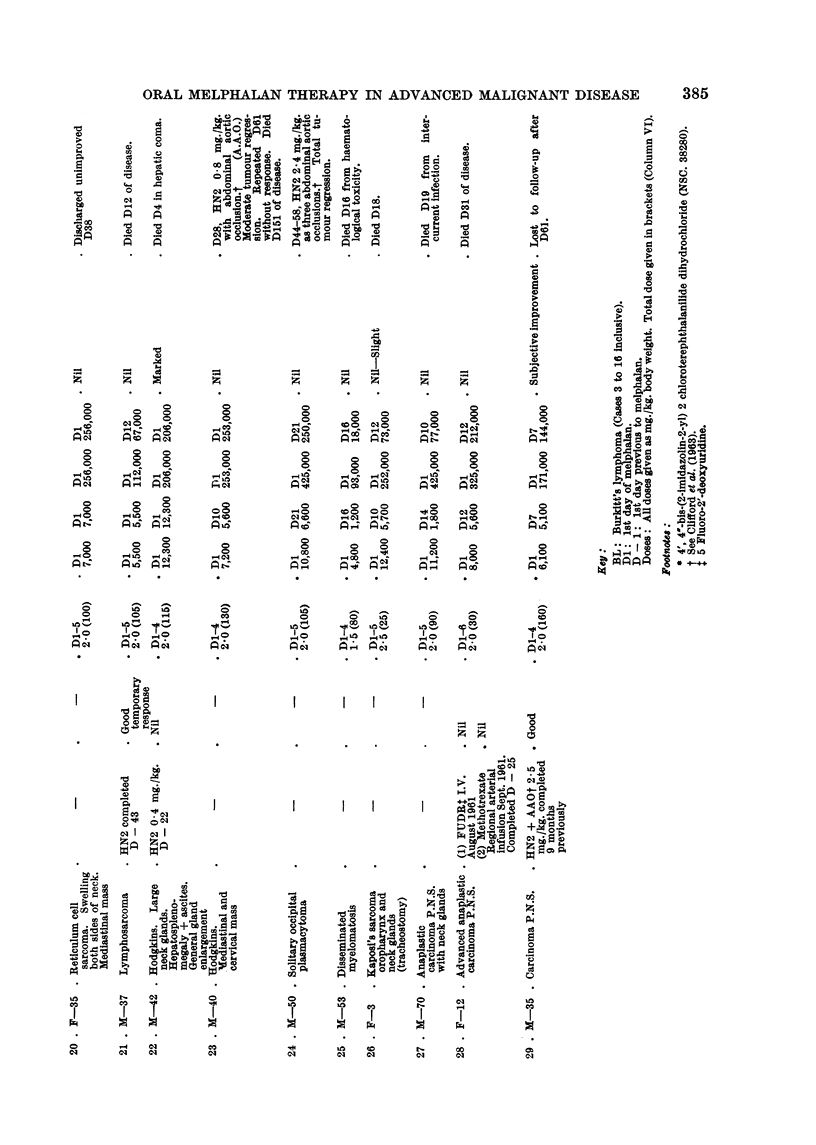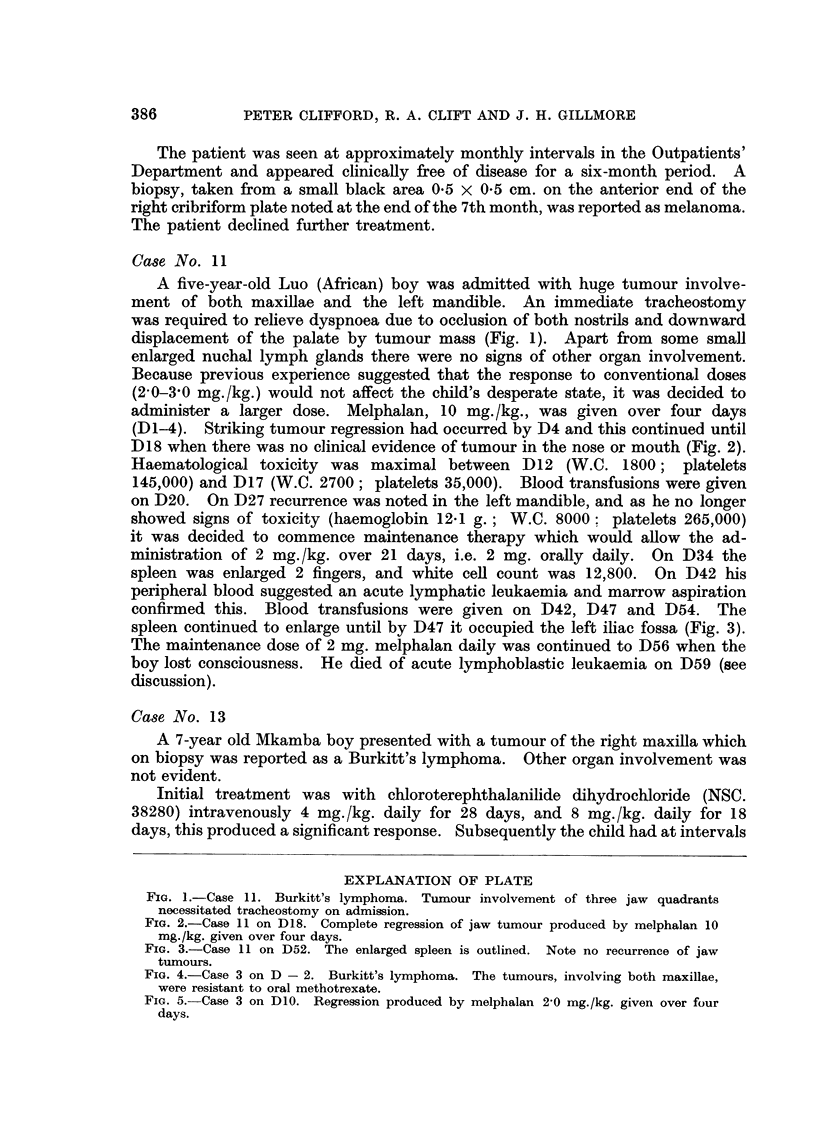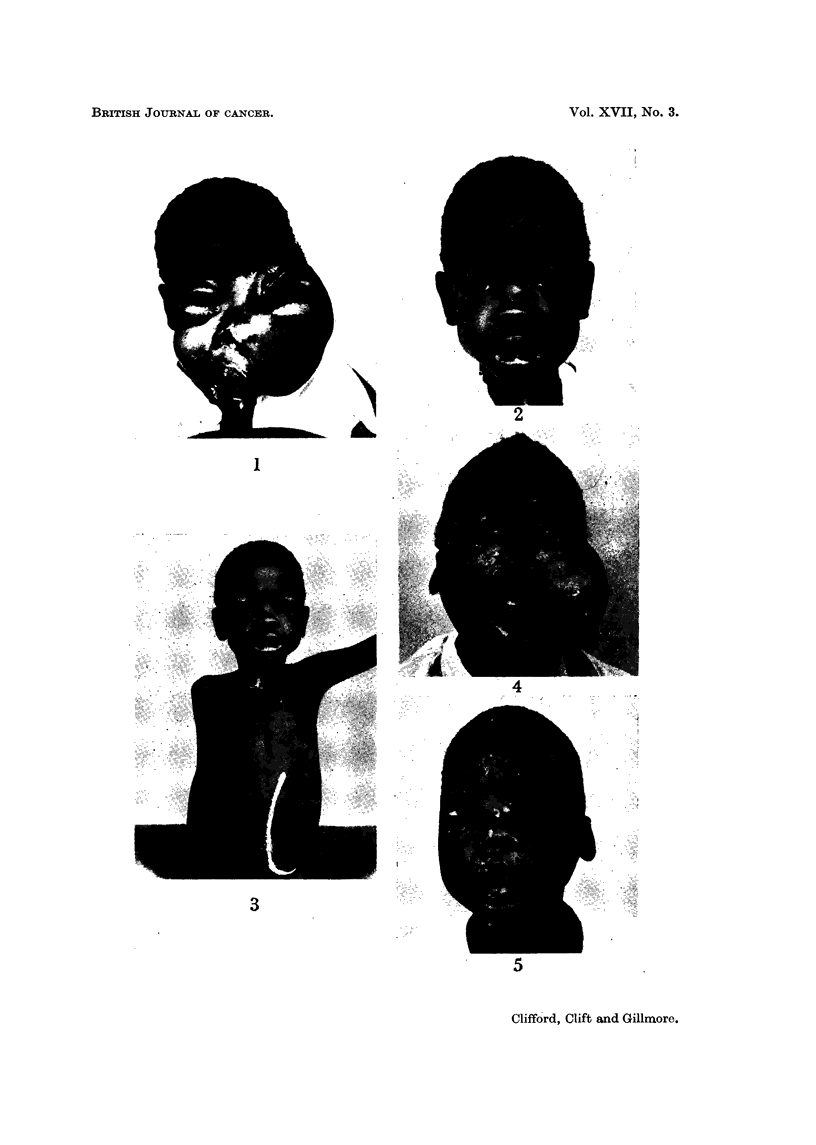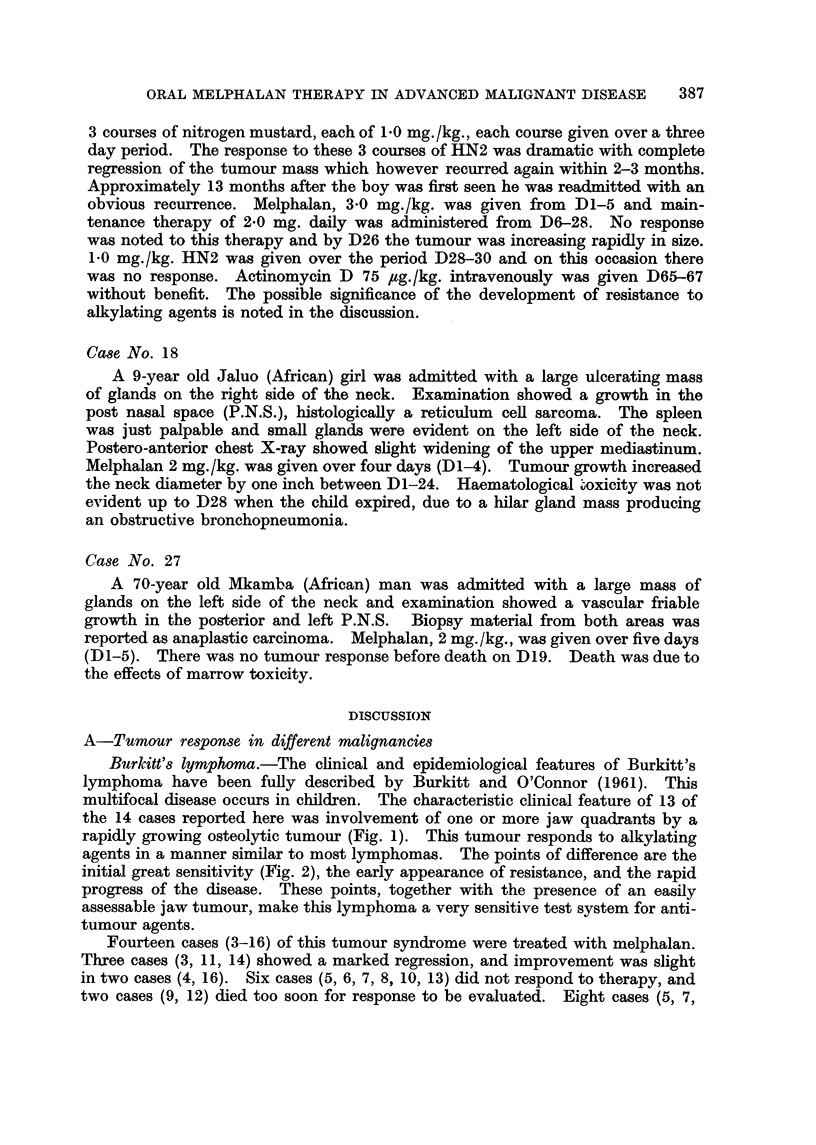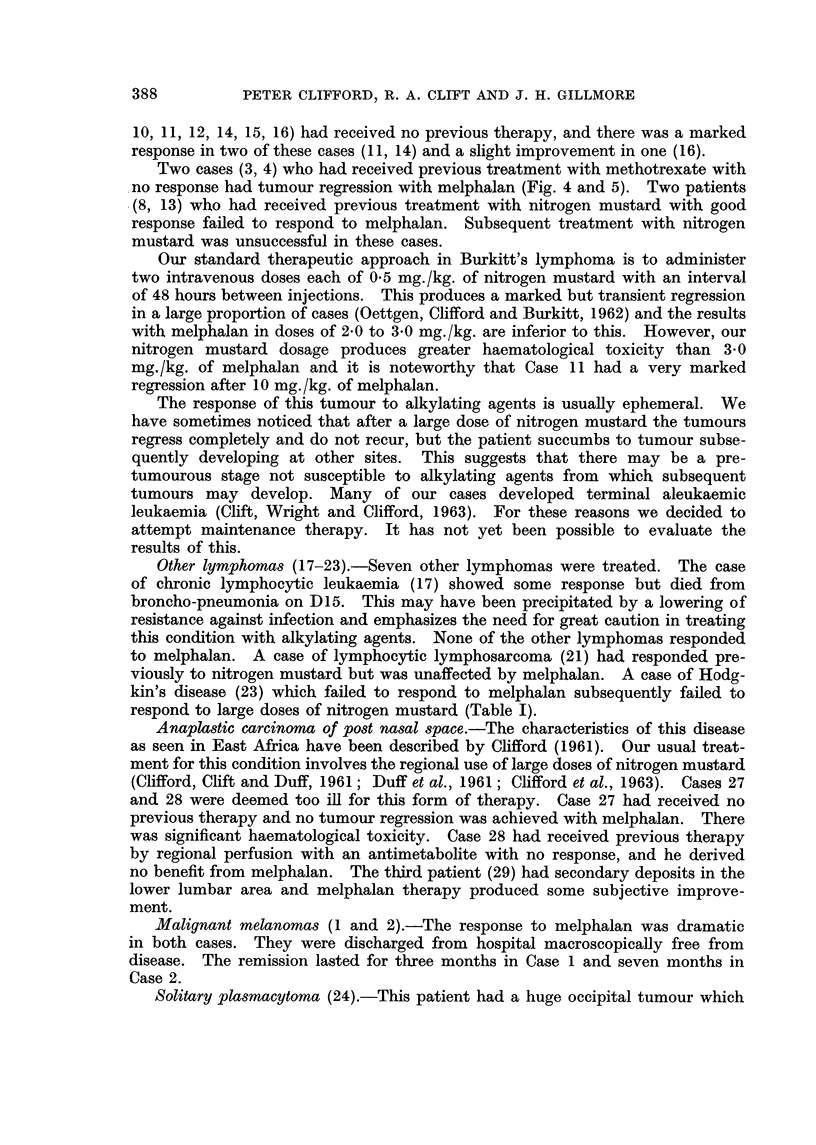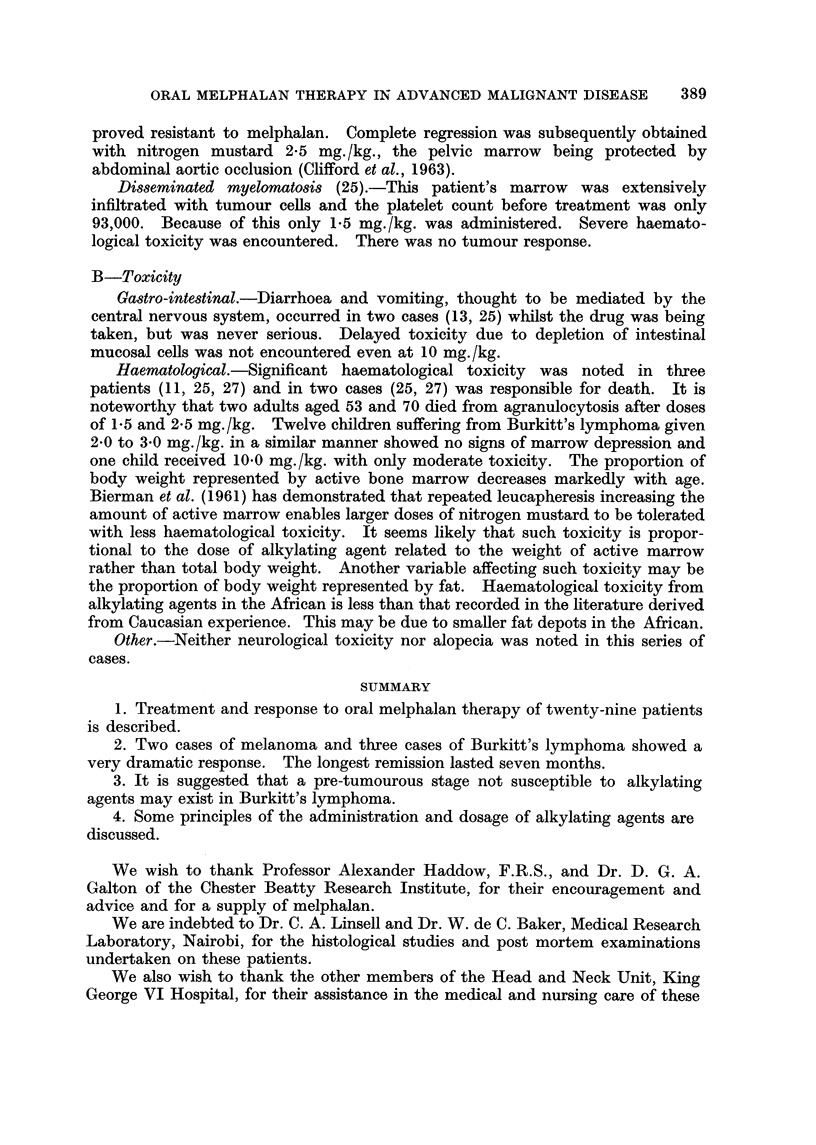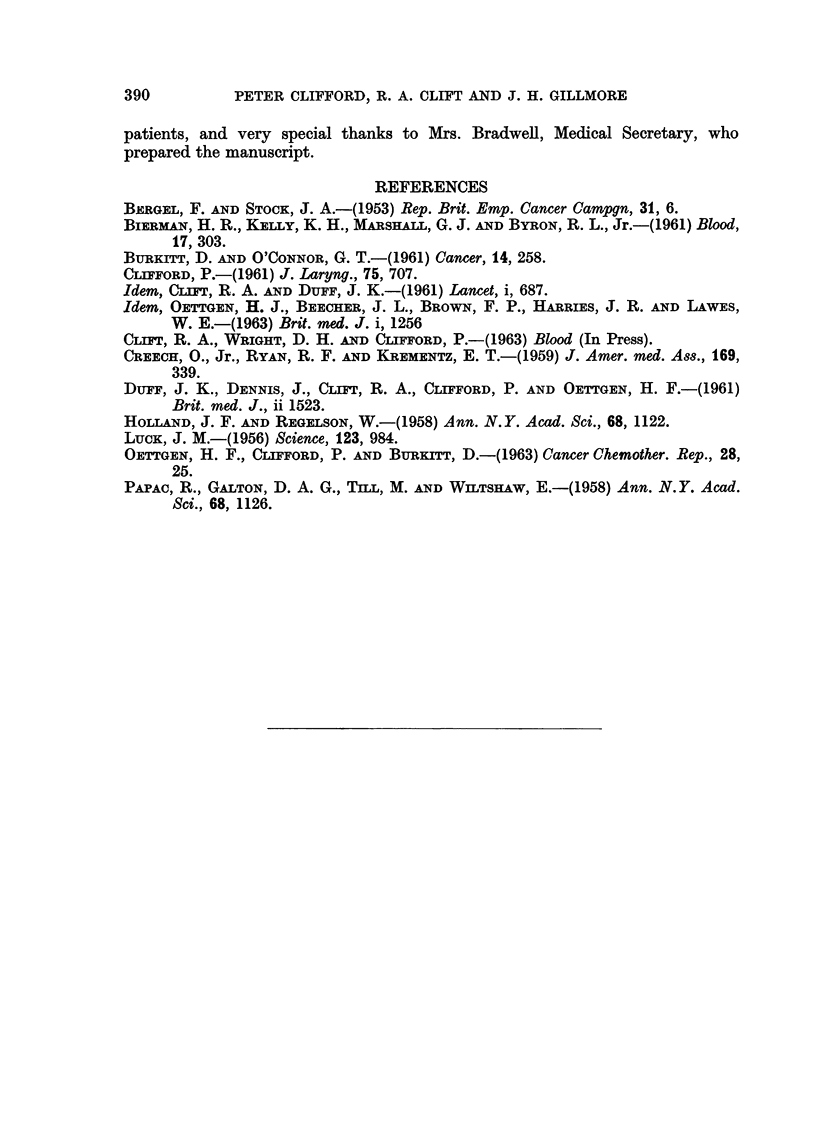# Oral Melphalan Therapy in Advanced Malignant Disease

**DOI:** 10.1038/bjc.1963.53

**Published:** 1963-09

**Authors:** Peter Clifford, R. A. Clift, J. H. Gillmore

## Abstract

**Images:**


### BRITISH JOURNAL OF CANCER

VOL. XVII SEPTEMBER, 1963 NO. 3

### ORAL MELPHALAN THERAPY IN ADVANCED MALIGNANT DISEASE

PETER CLIFFORD, R. A. CLIFT AND J. H. GILLMORE

*From the Department of Head and Neck Surgery, and The Medical Research Laboratory, King George VI Hospital, Nairobi, Kenya.*

Received for publication May 6, 1963.

MELPHALAN (Compound CB.3025), a nitrogen mustard derivative, was synthesized by Bergel and Stock in 1953. The compound is formed by the conjugation of the 2-chloroethyl amino group which is the active mustard configuration with the L isomer of the amino acid phenylalanine. The possibility exists that this compound may be incorporated into the intra-cellular pathways of protein synthesis, thus transporting the alkylating moiety of mustard into the cell. The compound has the following chemical structure :

*p*-di-2-chloroethylamino-L-phenylalanine.

Pharmacologically melphalan resembles nitrogen mustard, but it is well absorbed when administered by the oral route. The main toxic effects in man are similar to those of the other alkylating drugs ; thus the lymphoid and haemopoietic system, and the gastro-intestinal tract, are particularly vulnerable. Melphalan is insoluble in water but can be dissolved in an ethyl alcohol/propylene glycol mixture. Its half life in blood at 37° C. is 105–120 minutes.

Phenylalanine occupies a central place in the synthesis of melanin and it might be expected that melphalan would exert a major inhibitory effect on melanoma cells and Luck (1956) reported inhibition of the Harding-Passey melanoma in a small number of mice treated with this drug. Holland and Regelson (1958) treated 16 patients suffering from malignant melanoma with doses ranging from 0·55 mg./kg. to 2·22 mg./kg. and noted objective improvement in two cases. A series of cases treated by Papac *et al.* (1958) suggested that the drug might be of some value in Hodgkin's disease. Recent work (Speed, D. E., 1963, personal communication) suggests that about one-third of myeloma patients respond to melphalan; it has also been stated that response may occur in reticulum cell sarcoma, Kaposi's sarcoma, neuro- and fibrosarcoma and seminoma. Creech, Ryan and Krementz (1959) have administered melphalan by isolation perfusion to patients with melanoma of the extremities.

### *Method of Therapy*

Melphalan was given orally. Twenty-seven patients received 2·0 to 3·0 mg./kg. over a 4–5 day period. Two cases (11, 12) received 10·0 mg./kg. over 4 days. After the initial treatment, maintenance therapy was given to four patients (11, 13, 14, 16) the aim being to administer 2 mg./kg. over a 21 day period.

### *Clinical Material*

| | | |
|---|---|---|
| Melanomas | | 2 cases |
| Lymphomas | Burkitt's lymphomas | 14 „ |
| | Chronic lymphocytic leukaemia | 1 „ |
| | Reticulum cell sarcoma | 3 „ |
| | Lymphosarcoma | 1 „ |
| | Hodgkin's disease | 2 „ |
| Myelomas | Solitary | 1 „ |
| | Generalized | 1 „ |
| Kaposi's sarcoma | | 1 „ |
| Anaplastic carcinoma of post nasal space | | 3 „ |

The relevant clinical and therapeutic details of the twenty-nine patients reported in this series are outlined in Table I.

### *Illustrative Case Reports*

*Case No.* 2

A Kikuyu woman, aged 36 years, admitted complaining of pain and swelling of right orbit, and right nasal obstruction. Examination showed a dark red tumour occupying right, middle and superior nasal meatus. The right eye was normal. A firm discrete mass was palpable, occupying the medial and superior parts of the right orbit, the globe was displaced downwards and outwards. The right preauricular gland was enlarged, measuring 1 cm. × 1 cm. There was no evidence of further spread of the disease.

*Treatment.*—The right antro-ethmoidal area was explored using a lateral approach. The tumour was found to invade extensively the right orbit, which was exenterated together with the right ethmoidal labyrinth and frontal sinus, removal was incomplete about the bony floor of the right anterior cranial fossa. Histologically the tumour was a melanotic melanoma. Melphalan therapy was instituted on the fifth post-operative day (D1) as 2·0 mg./kg. (D1–5). Two further courses of melphalan 2·0 mg./kg. were administered from D40–44 and from D69–73. The relevant haematological findings are noted in Table I.

*Response.*—By D16 there was an obvious decrease in the size of the tumour and the preauricular gland. Improvement continued up to and after the second course of melphalan. Biopsies were taken on D51 from areas previously known to be involved by tumour and these were histologically negative. The preauricular gland was no longer palpable. The third course of melphalan was given because of the nature of the original growth.

TABLE I.—*Relevant Clinical and Therapeutic Details of Patients Reported in the Series.*

| I | II | III | IV | V | VI | VII | | | | VIII | IX |
|---|---|---|---|---|---|---|---|---|---|---|---|
| | | | | | | Haematological examination Leucocyte and platelet counts | | | | | |
| Case No. | Sex and age | Disease | Previous therapy and dates | Response | Mephalan therapy | Initial W.C. | Lowest W.C. | Initial platelet | Lowest platelet | Tumour response | Remarks |
| 1 | M—50 | Melanoma left maxilla (inf. turb.) | — | — | D1–4<br>2·0 (120) | D1<br>9,000 | D9<br>2,800 | D1<br>300,000 | D15<br>94,000 | Discharged macroscopically free of disease | Died D90. Secondary deposits in brain. |
| 2 | F—36½ | Malignant melanoma right antro-ethmoidal area and orbit | Incomplete surgical excision | — | D1–5<br>2·0 (85)<br>D40–44<br>2·0 (90)<br>D69–73<br>2·0 (90) | D1<br>9,600<br>D40<br>6,300<br>D68<br>7,600 | D14<br>4,200<br>D54<br>3,700<br>D83<br>2,800 | D1<br>266,000<br>D40<br>202,000<br>D68<br>281,000 | D14<br>121,000<br>D56<br>135,000<br>D83<br>76,000 | Good<br>Complete regression. No tumour evident histologically<br>Complete regression. No tumour evident histologically | Complete regression of disease for 6 months: Seen in O.P. Recurrence noted end of 7th month. |
| 3 | M—7 | BL. Both maxillae | Oral methotrexate 2·5–10 mg. daily completed D − 33 | Nil | D1–4<br>2·0 (35) | D2<br>18,500 | D15<br>4,750 | D2<br>212,000 | D16<br>97,000 | Marked regression | Regression lasted 2 weeks. Died D42 from disease. |
| 4 | F—7 | BL. Right maxilla and mandible | Oral methotrexate 2·5–10 mg. daily completed D −15 | Nil<br>Nil | D1–4<br>2·0 (35)<br>D62–65<br>2·0 (30) | D1<br>6,700<br>D62<br>14,000 | D20<br>2,400<br>D70<br>2,500 | D1<br>365,000<br>D62<br>275,000 | D20<br>190,000<br>D70<br>218,000 | Slight regression<br>No response | Regression lasted 8 days. Developed measles D10.<br>Died D93 from disease. |
| 5 | F—13 | BL. Right mandible | — | — | D1–5<br>2·0 (45) | D1<br>14,750 | D15<br>3,800 | D1<br>615,000 | D17<br>385,000 | Nil | Died D27 from disease. |
| 6 | M—5 | BL. Left maxilla | Actinomycin D. Cytoxan completed D − 25 | Nil | D1–6<br>2·0 (27·5) | D1<br>4,000 | D6<br>3,000 | D1<br>440,000 | D11<br>108,000 | Nil • | Moribund D27 from disease. Removed by parents to die at home. |
| 7 | F—6 | BL. Right mandible | — | — | D1–4<br>2·5 (40) | D1<br>5,900 | D17<br>2,300 | D1<br>196,000 | D20<br>144,000 | Nil | Died D49 from disease. |
| 8 | M—3½ | BL. Left mandible and maxilla | HN2 1·0 mg./kg. from D − 12 to D − 10 | Marked regression | D1–5<br>2·0 (30) | D1<br>3,200 | D1<br>3,200 | D1<br>130,000 | D1<br>130,000 | Nil | Because of rapid regrowth of tumour HN2 1 mg./kg. given without response on D16. Died D47 from disease. |
| 9 | M—3 | BL. Left maxilla | 6-Mercaptopurine 2·5 mg./kg. + Prednisone 10 mg. daily completed D − 13 | Nil | D1–5<br>3·0 (50) | D1<br>9,600 | D8<br>8,100 | D1<br>175,000 | D1<br>175,000 | Nil | Died D8. ST and BT malaria (pneumonia) and disease. |
| 10 | F—5 | BL. Right maxilla and mandible | — | — | D1–4<br>2·0 (35) | D1<br>7,100 | D9<br>3,800 | D1<br>280,000 | D9<br>112,000 | Nil | Patient commenced treatment with methotrexate 10 mg. orally daily D25–D55 without response. Died D63 from disease. |
| 11 | M—5 | BL. Both Maxillae and left mandible | — | — | D1–4<br>10·0 (105)<br>Maintenance daily dose 2 mg. D27–D56 | D1<br>7,100 | D12<br>1,800 | D1<br>285,000 | D17<br>35,000 | Total regression of maxillary and mandibular tumours by D18 | Overt acute lymphoblastic leukaemia on D42. Died of this disease on D59. |

TABLE I.—(*Continued*).

| I | II | III | IV | V | VI | VII | | | | VIII | IX |
|---|---|---|---|---|---|---|---|---|---|---|---|
| | | | | | | Haematological examination Leucocyte and platelet counts | | | | | |
| Case No. | Sex and age | Disease | Previous therapy and dates | Response | Melphalan therapy | Initial W.C. | Lowest W.C. | Initial platelet | Lowest platelet | Tumour response | Remarks |
| 12 | M—5 | BL. Right orbit and maxilla | — | — | D1–4<br>10·0 (105) | D1<br>7,600 | D1<br>7,600 | D1<br>375,000 | D6<br>260,000 | Nil | Died D6. PM—Very extensive involvement of anterior cranial fossa by tumour. |
| 13 | M—7 | BL. Right maxilla | TIDE Feb. 1962*<br>HN2 August 1962<br>HN2 November 1962<br>HN2 January 1963<br>completed D − 25 | Good response<br>,, ,,<br>,, ,,<br>,, ,, | D1–5<br>3·0 (35)<br>Maintenance daily dose 2·0 mg. D6–28 | D1<br>5,900 | D5<br>3,000<br>D36<br>1,900 | D1<br>270,000 | D13<br>185,000<br>D45<br>35,000 | Nil<br>Despite severe haematological toxicity tumour continued to grow | D28–30 had 1·0 mg./kg. HN2 without response.<br>D65–67. Actinomycin D 75 μg./kg. without response. |
| 14 | M—14 | BL. Left maxilla | — | — | D1–6<br>2·5 (120)<br>D31–37<br>2·5 (125)<br>D72–77<br>3·0 (160)<br>Maintenance daily dose 5 mg. D87–146 | D1<br>8,100<br>D31<br>8,300<br>D72<br>9,100 | D12<br>5,300<br>D48<br>3,600<br>D90<br>4,000 | D1<br>183,000<br>D31<br>260,000<br>D72<br>250,000 | D12<br>126,000<br>D48<br>130,000<br>D98<br>89,000 | Marked regression<br>Marked regression<br>Tumour continued to increase to D90 and thereafter.<br>Marked regression | Tumour growth recommenced D23.<br>Tumour growth recommenced D60.<br><br>Regression maintained to D147, when patient discharged at parents' request. Readmitted D169 with recurrence. |
| 15 | M—5 | BL. Right maxilla | — | — | D1–5<br>2·5 (35) | D1<br>15,600 | D12<br>8,700 | D1<br>387,000 | D12<br>294,000 | Nil | Died D24 of disease. |
| 16 | M—6 | BL. Abdomen. Main tumour mass on PM. about caecum | — | — | D1–5<br>3·0 (50)<br>D13–27<br>Maintenance daily dose 2 mg. | D1<br>6,200 | D6<br>2,500 | D1<br>330,000 | D6<br>85,000 | Improvement in lower limb oedema and ascites | Died D27 of disease. |
| 17 | M—60 | Chronic lymphocytic leukaemia with polyadenopathy | Chlorambucil 12 mg. daily completed D − 54 | WCC reduced from 400,000 to 5000. Little decrease in peripheral glands | D1–6<br>2·5 (112·5) | D1<br>81,800 | D12<br>2,500 | D1<br>177,000 | D12<br>147,000 | Slight regression of lymph glands | Died D15. Acute bronchopneumonia. |
| 18 | F—9 | Reticulum cell sarcoma P.N.S. Glands + + + | — | — | D1–4<br>2·0 (70) | D1<br>8,200 | D18<br>6,000 | D1<br>350,000 | D18<br>117,000 | Nil. Tumour growth increased the neck circumference by 1 in. while under treatment | Died D28. PM — right broncho-pneumonia due to obstruction by hilar mass. |
| 19 | F—40 | Reticulum cell sarcoma. Huge retropharyngeal tumour necessitating tracheostomy | — | — | D1–6<br>2·0 (100) | D1<br>11,000 | D12<br>5,700 | D1<br>270,000 | D14<br>223,000 | Nil | Discharged unimproved D38. |

| | | | | | | | | | | | |
|---|---|---|---|---|---|---|---|---|---|---|---|
| 20 | F—35 | Reticulum cell sarcoma. Swelling both sides of neck. Mediastinal mass | — | — | D1–5 2·0 (100) | D1 7,000 | D1 7,000 | D1 256,000 | D1 256,000 | Nil | Discharged unimproved D38 |
| 21 | M—37 | Lymphosarcoma | HN2 completed D − 43 | Good temporary response | D1–5 2·0 (105) | D1 5,500 | D1 5,500 | D1 112,000 | D12 67,000 | Nil | Died D12 of disease. |
| 22 | M—42 | Hodgkins. Large neck glands. Hepatosplenomegaly + ascites. General gland enlargement | HN2 0·4 mg./kg. D − 22 | Nil | D1–4 2·0 (115) | D1 12,300 | D1 12,300 | D1 206,000 | D1 206,000 | Marked | Died D4 in hepatic coma. |
| 23 | M—40 | Hodgkins. Mediastinal and cervical mass | — | — | D1–4 2·0 (130) | D1 7,200 | D10 5,600 | D1 253,000 | D1 253,000 | Nil | D28, HN2 0·8 mg./kg. with abdominal aortic occlusion.† (A.A.O.) Moderate tumour regression. Repeated D61 without response. Died D151 of disease. |
| 24 | M—50 | Solitary occipital plasmacytoma | — | — | D1–5 2·0 (105) | D1 10,800 | D21 6,600 | D1 425,000 | D21 250,000 | Nil | D44–58, HN2 2·4 mg./kg. as three abdominal aortic occlusions.† Total tumour regression. |
| 25 | M—53 | Disseminated myelomatosis | — | — | D1–4 1·5 (80) | D1 4,800 | D16 1,200 | D1 93,000 | D16 18,000 | Nil | Died D16 from haematological toxicity. |
| 26 | F—3 | Kaposi's sarcoma oropharynx and neck glands (tracheostomy) | — | — | D1–5 2·5 (25) | D1 12,400 | D10 5,700 | D1 252,000 | D12 73,000 | Nil—Slight | Died D18. |
| 27 | M—70 | Anaplastic carcinoma P.N.S. with neck glands | — | — | D1–5 2·0 (90) | D1 11,200 | D14 1,800 | D1 425,000 | D10 77,000 | Nil | Died D19 from intercurrent infection. |
| 28 | F—12 | Advanced anaplastic carcinoma P.N.S. | (1) FUDR‡ I.V. August 1961<br>(2) Methotrexate Regional arterial infusion Sept. 1961. Completed D − 25 | Nil<br>Nil | D1–6 2·0 (30) | D1 8,000 | D12 5,600 | D1 325,000 | D12 212,000 | Nil | Died D31 of disease. |
| 29 | M—35 | Carcinoma P.N.S. | HN2 + AAO† 2·5 mg./kg. completed 9 months previously | Good | D1–4 2·0 (160) | D1 6,100 | D7 5,100 | D1 171,000 | D7 144,000 | Subjective improvement | Lost to follow-up after D61. |

***Key :***

BL : Burkitt's lymphoma (Cases 3 to 16 inclusive).

D1 : 1st day of melphalan.

D − 1 : 1st day previous to melphalan.

Doses : All doses given as mg./kg. body weight. Total dose given in brackets (Column VI).

***Footnotes :***

* 4′, 4″-bis-(2-imidazolin-2-yl) 2 chloroterephthalanilide dihydrochloride (NSC. 38280).

† See Clifford *et al.* (1963).

‡ 5 Fluoro-2′-deoxyuridine.

The patient was seen at approximately monthly intervals in the Outpatients' Department and appeared clinically free of disease for a six-month period. A biopsy, taken from a small black area 0·5 × 0·5 cm. on the anterior end of the right cribriform plate noted at the end of the 7th month, was reported as melanoma. The patient declined further treatment.

*Case No.* 11

A five-year-old Luo (African) boy was admitted with huge tumour involvement of both maxillae and the left mandible. An immediate tracheostomy was required to relieve dyspnoea due to occlusion of both nostrils and downward displacement of the palate by tumour mass (Fig. 1). Apart from some small enlarged nuchal lymph glands there were no signs of other organ involvement. Because previous experience suggested that the response to conventional doses (2·0–3·0 mg./kg.) would not affect the child's desperate state, it was decided to administer a larger dose. Melphalan, 10 mg./kg., was given over four days (D1–4). Striking tumour regression had occurred by D4 and this continued until D18 when there was no clinical evidence of tumour in the nose or mouth (Fig. 2). Haematological toxicity was maximal between D12 (W.C. 1800; platelets 145,000) and D17 (W.C. 2700; platelets 35,000). Blood transfusions were given on D20. On D27 recurrence was noted in the left mandible, and as he no longer showed signs of toxicity (haemoglobin 12·1 g.; W.C. 8000; platelets 265,000) it was decided to commence maintenance therapy which would allow the administration of 2 mg./kg. over 21 days, i.e. 2 mg. orally daily. On D34 the spleen was enlarged 2 fingers, and white cell count was 12,800. On D42 his peripheral blood suggested an acute lymphatic leukaemia and marrow aspiration confirmed this. Blood transfusions were given on D42, D47 and D54. The spleen continued to enlarge until by D47 it occupied the left iliac fossa (Fig. 3). The maintenance dose of 2 mg. melphalan daily was continued to D56 when the boy lost consciousness. He died of acute lymphoblastic leukaemia on D59 (see discussion).

*Case No.* 13

A 7-year old Mkamba boy presented with a tumour of the right maxilla which on biopsy was reported as a Burkitt's lymphoma. Other organ involvement was not evident.

Initial treatment was with chloroterephthalanilide dihydrochloride (NSC. 38280) intravenously 4 mg./kg. daily for 28 days, and 8 mg./kg. daily for 18 days, this produced a significant response. Subsequently the child had at intervals

---

EXPLANATION OF PLATE

FIG. 1.—Case 11. Burkitt's lymphoma. Tumour involvement of three jaw quadrants necessitated tracheostomy on admission.

FIG. 2.—Case 11 on D18. Complete regression of jaw tumour produced by melphalan 10 mg./kg. given over four days.

FIG. 3.—Case 11 on D52. The enlarged spleen is outlined. Note no recurrence of jaw tumours.

FIG. 4.—Case 3 on D − 2. Burkitt's lymphoma. The tumours, involving both maxillae, were resistant to oral methotrexate.

FIG. 5.—Case 3 on D10. Regression produced by melphalan 2·0 mg./kg. given over four days.

1

2

3

4

5

3 courses of nitrogen mustard, each of 1·0 mg./kg., each course given over a three day period. The response to these 3 courses of HN2 was dramatic with complete regression of the tumour mass which however recurred again within 2–3 months. Approximately 13 months after the boy was first seen he was readmitted with an obvious recurrence. Melphalan, 3·0 mg./kg. was given from D1–5 and maintenance therapy of 2·0 mg. daily was administered from D6–28. No response was noted to this therapy and by D26 the tumour was increasing rapidly in size. 1·0 mg./kg. HN2 was given over the period D28–30 and on this occasion there was no response. Actinomycin D 75 μg./kg. intravenously was given D65–67 without benefit. The possible significance of the development of resistance to alkylating agents is noted in the discussion.

*Case No.* 18

A 9-year old Jaluo (African) girl was admitted with a large ulcerating mass of glands on the right side of the neck. Examination showed a growth in the post nasal space (P.N.S.), histologically a reticulum cell sarcoma. The spleen was just palpable and small glands were evident on the left side of the neck. Postero-anterior chest X-ray showed slight widening of the upper mediastinum. Melphalan 2 mg./kg. was given over four days (D1–4). Tumour growth increased the neck diameter by one inch between D1–24. Haematological toxicity was not evident up to D28 when the child expired, due to a hilar gland mass producing an obstructive bronchopneumonia.

*Case No.* 27

A 70-year old Mkamba (African) man was admitted with a large mass of glands on the left side of the neck and examination showed a vascular friable growth in the posterior and left P.N.S. Biopsy material from both areas was reported as anaplastic carcinoma. Melphalan, 2 mg./kg., was given over five days (D1–5). There was no tumour response before death on D19. Death was due to the effects of marrow toxicity.

### DISCUSSION

### A—*Tumour response in different malignancies*

*Burkitt's lymphoma.*—The clinical and epidemiological features of Burkitt's lymphoma have been fully described by Burkitt and O'Connor (1961). This multifocal disease occurs in children. The characteristic clinical feature of 13 of the 14 cases reported here was involvement of one or more jaw quadrants by a rapidly growing osteolytic tumour (Fig. 1). This tumour responds to alkylating agents in a manner similar to most lymphomas. The points of difference are the initial great sensitivity (Fig. 2), the early appearance of resistance, and the rapid progress of the disease. These points, together with the presence of an easily assessable jaw tumour, make this lymphoma a very sensitive test system for anti-tumour agents.

Fourteen cases (3–16) of this tumour syndrome were treated with melphalan. Three cases (3, 11, 14) showed a marked regression, and improvement was slight in two cases (4, 16). Six cases (5, 6, 7, 8, 10, 13) did not respond to therapy, and two cases (9, 12) died too soon for response to be evaluated. Eight cases (5, 7,

10, 11, 12, 14, 15, 16) had received no previous therapy, and there was a marked response in two of these cases (11, 14) and a slight improvement in one (16).

Two cases (3, 4) who had received previous treatment with methotrexate with no response had tumour regression with melphalan (Fig. 4 and 5). Two patients (8, 13) who had received previous treatment with nitrogen mustard with good response failed to respond to melphalan. Subsequent treatment with nitrogen mustard was unsuccessful in these cases.

Our standard therapeutic approach in Burkitt's lymphoma is to administer two intravenous doses each of 0·5 mg./kg. of nitrogen mustard with an interval of 48 hours between injections. This produces a marked but transient regression in a large proportion of cases (Oettgen, Clifford and Burkitt, 1962) and the results with melphalan in doses of 2·0 to 3·0 mg./kg. are inferior to this. However, our nitrogen mustard dosage produces greater haematological toxicity than 3·0 mg./kg. of melphalan and it is noteworthy that Case 11 had a very marked regression after 10 mg./kg. of melphalan.

The response of this tumour to alkylating agents is usually ephemeral. We have sometimes noticed that after a large dose of nitrogen mustard the tumours regress completely and do not recur, but the patient succumbs to tumour subsequently developing at other sites. This suggests that there may be a pre-tumourous stage not susceptible to alkylating agents from which subsequent tumours may develop. Many of our cases developed terminal aleukaemic leukaemia (Clift, Wright and Clifford, 1963). For these reasons we decided to attempt maintenance therapy. It has not yet been possible to evaluate the results of this.

*Other lymphomas* (17–23).—Seven other lymphomas were treated. The case of chronic lymphocytic leukaemia (17) showed some response but died from broncho-pneumonia on D15. This may have been precipitated by a lowering of resistance against infection and emphasizes the need for great caution in treating this condition with alkylating agents. None of the other lymphomas responded to melphalan. A case of lymphocytic lymphosarcoma (21) had responded previously to nitrogen mustard but was unaffected by melphalan. A case of Hodgkin's disease (23) which failed to respond to melphalan subsequently failed to respond to large doses of nitrogen mustard (Table I).

*Anaplastic carcinoma of post nasal space.*—The characteristics of this disease as seen in East Africa have been described by Clifford (1961). Our usual treatment for this condition involves the regional use of large doses of nitrogen mustard (Clifford, Clift and Duff, 1961; Duff *et al.*, 1961; Clifford *et al.*, 1963). Cases 27 and 28 were deemed too ill for this form of therapy. Case 27 had received no previous therapy and no tumour regression was achieved with melphalan. There was significant haematological toxicity. Case 28 had received previous therapy by regional perfusion with an antimetabolite with no response, and he derived no benefit from melphalan. The third patient (29) had secondary deposits in the lower lumbar area and melphalan therapy produced some subjective improvement.

*Malignant melanomas* (1 and 2).—The response to melphalan was dramatic in both cases. They were discharged from hospital macroscopically free from disease. The remission lasted for three months in Case 1 and seven months in Case 2.

*Solitary plasmacytoma* (24).—This patient had a huge occipital tumour which proved resistant to melphalan. Complete regression was subsequently obtained with nitrogen mustard 2·5 mg./kg., the pelvic marrow being protected by abdominal aortic occlusion (Clifford *et al.*, 1963).

*Disseminated myelomatosis* (25).—This patient's marrow was extensively infiltrated with tumour cells and the platelet count before treatment was only 93,000. Because of this only 1·5 mg./kg. was administered. Severe haematological toxicity was encountered. There was no tumour response.

B—*Toxicity*

*Gastro-intestinal.*—Diarrhoea and vomiting, thought to be mediated by the central nervous system, occurred in two cases (13, 25) whilst the drug was being taken, but was never serious. Delayed toxicity due to depletion of intestinal mucosal cells was not encountered even at 10 mg./kg.

*Haematological.*—Significant haematological toxicity was noted in three patients (11, 25, 27) and in two cases (25, 27) was responsible for death. It is noteworthy that two adults aged 53 and 70 died from agranulocytosis after doses of 1·5 and 2·5 mg./kg. Twelve children suffering from Burkitt's lymphoma given 2·0 to 3·0 mg./kg. in a similar manner showed no signs of marrow depression and one child received 10·0 mg./kg. with only moderate toxicity. The proportion of body weight represented by active bone marrow decreases markedly with age. Bierman *et al.* (1961) has demonstrated that repeated leucapheresis increasing the amount of active marrow enables larger doses of nitrogen mustard to be tolerated with less haematological toxicity. It seems likely that such toxicity is proportional to the dose of alkylating agent related to the weight of active marrow rather than total body weight. Another variable affecting such toxicity may be the proportion of body weight represented by fat. Haematological toxicity from alkylating agents in the African is less than that recorded in the literature derived from Caucasian experience. This may be due to smaller fat depots in the African.

*Other.*—Neither neurological toxicity nor alopecia was noted in this series of cases.

SUMMARY

1. Treatment and response to oral melphalan therapy of twenty-nine patients is described.

2. Two cases of melanoma and three cases of Burkitt's lymphoma showed a very dramatic response. The longest remission lasted seven months.

3. It is suggested that a pre-tumourous stage not susceptible to alkylating agents may exist in Burkitt's lymphoma.

4. Some principles of the administration and dosage of alkylating agents are discussed.

We wish to thank Professor Alexander Haddow, F.R.S., and Dr. D. G. A. Galton of the Chester Beatty Research Institute, for their encouragement and advice and for a supply of melphalan.

We are indebted to Dr. C. A. Linsell and Dr. W. de C. Baker, Medical Research Laboratory, Nairobi, for the histological studies and post mortem examinations undertaken on these patients.

We also wish to thank the other members of the Head and Neck Unit, King George VI Hospital, for their assistance in the medical and nursing care of these patients, and very special thanks to Mrs. Bradwell, Medical Secretary, who prepared the manuscript.

### REFERENCES

BERGEL, F. AND STOCK, J. A.—(1953) *Rep. Brit. Emp. Cancer Campgn*, **31**, 6.

BIERMAN, H. R., KELLY, K. H., MARSHALL, G. J. AND BYRON, R. L., Jr.—(1961) *Blood*, **17**, 303.

BURKITT, D. AND O'CONNOR, G. T.—(1961) *Cancer*, **14**, 258.

CLIFFORD, P.—(1961) *J. Laryng.*, **75**, 707.

*Idem*, CLIFT, R. A. AND DUFF, J. K.—(1961) *Lancet*, i, 687.

*Idem*, OETTGEN, H. J., BEECHER, J. L., BROWN, F. P., HARRIES, J. R. AND LAWES, W. E.—(1963) *Brit. med. J.* i, 1256

CLIFT, R. A., WRIGHT, D. H. AND CLIFFORD, P.—(1963) *Blood* (In Press).

CREECH, O., Jr., RYAN, R. F. AND KREMENTZ, E. T.—(1959) *J. Amer. med. Ass.*, **169**, 339.

DUFF, J. K., DENNIS, J., CLIFT, R. A., CLIFFORD, P. AND OETTGEN, H. F.—(1961) *Brit. med. J.*, ii 1523.

HOLLAND, J. F. AND REGELSON, W.—(1958) *Ann. N.Y. Acad. Sci.*, **68**, 1122.

LUCK, J. M.—(1956) *Science*, **123**, 984.

OETTGEN, H. F., CLIFFORD, P. AND BURKITT, D.—(1963) *Cancer Chemother. Rep.*, **28**, 25.

PAPAC, R., GALTON, D. A. G., TILL, M. AND WILTSHAW, E.—(1958) *Ann. N.Y. Acad. Sci.*, **68**, 1126.